# Comparing the structure-function relationship between the visual fields measured with variational Bayes linear regression and SITA standard

**DOI:** 10.1371/journal.pone.0282638

**Published:** 2023-03-06

**Authors:** Ryo Asaoka, Hiroshi Murata, Yuri Fujino, Shuichiro Aoki, Kazunori Hirasawa, Nobuyuki Shoji

**Affiliations:** 1 Department of Ophthalmology, The University of Tokyo, Tokyo, Japan; 2 Department of Ophthalmology, Seirei Hamamatsu General Hospital, Hamamatsu, Japan; 3 Seirei Christopher University, Hamamatsu, Shizuoka, Japan; 4 The Graduate School for the Creation of New Photonics Industries, Hamamatsu, Shizuoka Japan; 5 Department of Ophthalmology, National Center for Global health and Medicine, Tokyo, Japan; 6 Department of Ophthalmology, Shimane University, Matsue, Shimane, Japan; 7 Department of Ophthalmology, Kitasato University School of Medicine, Sagamihara, Kanagawa, Japan; The University of Iowa, UNITED STATES

## Abstract

**Purpose:**

We recently constructed an algorithm to measure visual field (VF) using the variational Bayes linear regression (VBLR). This algorithm enabled a faster VF measurement than the Swedish interactive thresholding algorithm (SITA) standard while maintaining the test-retest reproducibility (Murata H, et al. Br J Ophthalmol 2021). The current study aimed to compare the structure-function relationship between the SITA standard and VBLR.

**Method:**

In 78 eyes of 56 patients with primary open-angle glaucoma, VF measurements were conducted using both SITA standard and VBLR VF, as well as spectral-domain optical coherence tomography. The structure-function relationship was investigated between visual sensitivity and circumpapillary retinal nerve fiber layer in the whole VF. This analysis was repeated for each of the 12 sectors (30 degrees). The strength of the structure-function relationship was evaluated using the second-order bias-corrected Akaike Information Criterion (AICc) index.

**Result:**

In the whole VF, AICc values of SITA standard and VBLR were 601.6 and 597.3, respectively. The relative likelihood that VBLR had a better structure-function relationship than the SITA standard was 88.2% (when the entire field was averaged) or 99.9% (when all test points were analyzed in the pointwise manner). With the sector-wise analysis, SITA standard had a better structure-function relationship than VBLR in 1 sector (Superior sector in the retina), whereas VBLR had a better structure-function relationship than SITA standard in 4 sectors (Supero-Nasal, Infero-Nasal, Inferior, and Infero-Temporal sectors) with >95% relative likelihood.

**Conclusion:**

Although it depends on locations and similar between SITA standard and VBLR-VF, but VBLR-VF had a better structure-function relationship than the SITA standard overall.

## Introduction

Without a doubt, static automated perimetry is the gold standard for diagnosing and monitoring progression in glaucoma. The Swedish interactive thresholding algorithm (SITA) standard program [1, 2] is one of the most widely used methods to measure VF from the early days of the Humphry Field Analyzer (HFA, Carl Zeiss Meditec, Dublin, USA), enabling the reduction of measurement duration by up to 50% [3] compared to the former Full Threshold program. This advancement was achieved by continuously updating the iterative maximum posterior probability estimation of the threshold in a Bayesian prior model and interrupting testing at each tested location at predetermined levels of test certainty. This has also led to an improvement in test-retest reproducibility, probably due to the avoidance of patients’ fatigue [4].

We previously developed a method to predict future VF deterioration based on the variational Bayes linear regression (VBLR) model [5, 6]. The VBLR model is a method to predict VF progression much more accurately than the conventional ordinary least square linear regression model by taking into account the spatial and temporal patterns of VF damage using the variational Bayes statistic [5, 6]. This method is then used to create an algorithm to measure VF faster than SITA (by 30 or 40 (approximately -10%) with a 4–2 dB double cross staircase method [7] or by 30 or 80 seconds (approximately -10% or -30%) with a single staircase method [8] without sacrificing the test-retest reproducibility; the algorithms are now implemented in the perimetry of KOWA AP7700 (Kowa Co. Ltd, Nagoya Japan). Although these advances were made without compromising the test-retest reproducibility of the measured sensitivity,　these two methods have not been compared in terms of the structure-function relationship. The goal of this study was to prospectively compare the structure-function relationships of the visual sensitivities and optical coherence tomography (OCT) measured circumpapillary retinal nerve fiber layer (cpRNFL) thickness, between VBLR and SITA standard.

## Method

The Research Ethics Committee of the Graduate School of Medicine and the Faculty of Medicine at the University of Tokyo (#10907) approved this study. Patients provided written consent for their information to be stored in the hospital database and used for research purposes. This research was carried out in accordance with the principles of the Helsinki Declaration.

### Subjects

This study included the 78 eyes of 56 patients with primary open-angle glaucoma (POAG), followed up at the Tokyo University Hospital. Eyes were inherited from our previous study where OCT measurement was available [7]. When repeated VF measurements were taken, the data obtained on the same date with OCT was used. The diagnosis of POAG, as well as the inclusion and exclusion criteria, were based on our previous research; in short, all of the eyes had a glaucomatous VF defect without any ocular pathology other than POAG [6].

### VF measurement with SITA standard

The SITA standard measurement was carried out using the HFA 24–2 test and standard Goldmann III stimulus size.

### VF measurement with VBLR-VF

VF measurement algorithm using VBLR was performed using the KOWA AP-7700. As previously described in our prior publications [8, 9], VBLR-VF can avoid redundant target presentation by optimizing the starting stimulus intensities through the VF sensitivity prediction in the VBLR model. Furthermore, the VF measurement was terminated when the sensitivity was estimated to be sufficiently accurate in the VBLR model using Bayesian likelihood estimation. This measurement was conducted without referring to each patient’s past VF measurement, assuming that the initial VF measurement for the patient, i.e., the prior model and starting stimulus intensities were calculated as the first-time measurement in each measurement. A 4–2 dB double cross staircase method was used, which means that 4 dB was applied until the first reversal, then 2 dB steps until the second. KOWA-AP7700 has been equipped with another VF measurement algorithm of ‘Quick1’, which uses a 3 dB single staircase method. VBLR-VF was developed to achieve a fast VF measurement without ceasing the 4–2 dB double cross staircase used in SITA standard.

VF measurements with SITA-Standard and VBLR-VF were conducted twice in random order on a same day, with an interval of 5 minutes. Only reliable VFs were used in the analyses, defined as a fixation loss rate <33%, a false-positive rate <33% and an FN rate <33%.

### OCT imaging

Within 3 months of the VF measurements, OCT data were obtained using the RS-3000 (Nidek Co.ltd., Aichi, Japan). Measurements were performed after pupillary dilation with mydriatic drug (1% tropicamide), and OCT imaging was performed using the protocol raster-scan, and a 6.0 × 6.0 mm^2^ area (512 × 128 pixels). Data with a signal strength index <7 were excluded as recommended by the manufacturer. Images affected by eye movements, involuntary blinking, or saccades were also carefully excluded. The cpRNFL thickness was measured at 1024 points (approximately every 0.35 degrees) from the most temporal side toward the clockwise direction (9-o’clock position, right eye: 0 degrees). The magnification effect was corrected according to the manufacturer-provided formula (a modified Littman’s equation) [10, 11], which is based on measured refractive error, corneal radius, and axial length. Optic disc was divided into 12 30-degree sectors, and sectorial cpRNFL thickness was calculated as the mean value of 30 degrees from 0 degrees; sector 1: 0 to 30 degrees, sector 2: 30 to 60 degrees, sector 3: 60 to 90 degrees, sector 4: 90 to 120 degrees, sector 5: 120 to 150 degrees, sector 6: 150 to 180 degrees, sector 7: 180 to 210 degrees, sector 8: 210 to 240 degrees, sector 9: 240 to 270 degrees, sector 10: 270 to 300 degrees, sector 11: 300 to 330 degrees, and sector 12: 330 to 360 degrees.

### Axial length measurement

All patients had axial length (AL) measured using the IOL Master (Ver4) (Carl Zeiss Meditec), by a well-trained examiner.

### Statistical analysis

First, the structure-function relationship was investigated between the average of entire cpRNFL thickness (dependent variable) and mean threshold value in whole VF for each of SITA standard and VBLR-VF, using the linear mixed model where the random effect was patients. The linear mixed model is equivalent to ordinary linear regression in that the model describes the relationship between the predictor variables and a single outcome variable. However, standard linear regression analysis makes the assumption that all observations are independent of each other. In this study, measurements were nested within subjects and thus dependent on one another. Ignoring this grouping of the measurements will result in the underestimation of standard errors of regression coefficients. The linear mixed model adjusts for the hierarchical structure of the data, modeling in a way in which measurements are grouped within subjects to reduce the possible bias derived from the nested structure of data [12, 13]. The second-order bias-corrected Akaike Information Criterion (AICc) index was used to compare the structure-function relationship for each measurement. The AIC is an established statistical measure used to evaluate the relationship between variables, and the AICc is a corrected type of the AIC, which provides an accurate estimation even when the sample size is small [14]. Any magnitude reduction in AICc indicates an improved model, but it is possible to estimate the likelihood that one model is the model that minimizes ‘‘information loss.” Suppose that there are *n* candidate models and the AICc values of those models are AIC*1*, AIC*2*, AIC*3*,…, AIC*n*. Let AIC*min* be the minimum of those values. Then exp((AIC*min* _ AIC*i*)/2) can be interpreted as the relative probability that the *i*th model minimizes the information loss [15, 16]. Thus, the relative probabilities were calculated among the models. There is no method to calculate the correlation coefficient with a linear mixed mode. Instead, in the current study, marginal and conditional R^2^ values (mR^2^ and cR^2^, respectively) were calculated following the method by Nakagawa et al. [17] The former is the variance only by the fixed effects, and the latter is that by both the fixed and random effects.

Followingly, the structure-function relationship was investigated between pointwise visual sensitivities (52 test points, fixed effect) and corresponding sectorial cpRNFL thicknesses, using the linear mixed model where the random effect was patients and test points (dependent variable was cpRNFL thickness), and compared between SITA standard and VBLR-VF.

**Fig 1:**
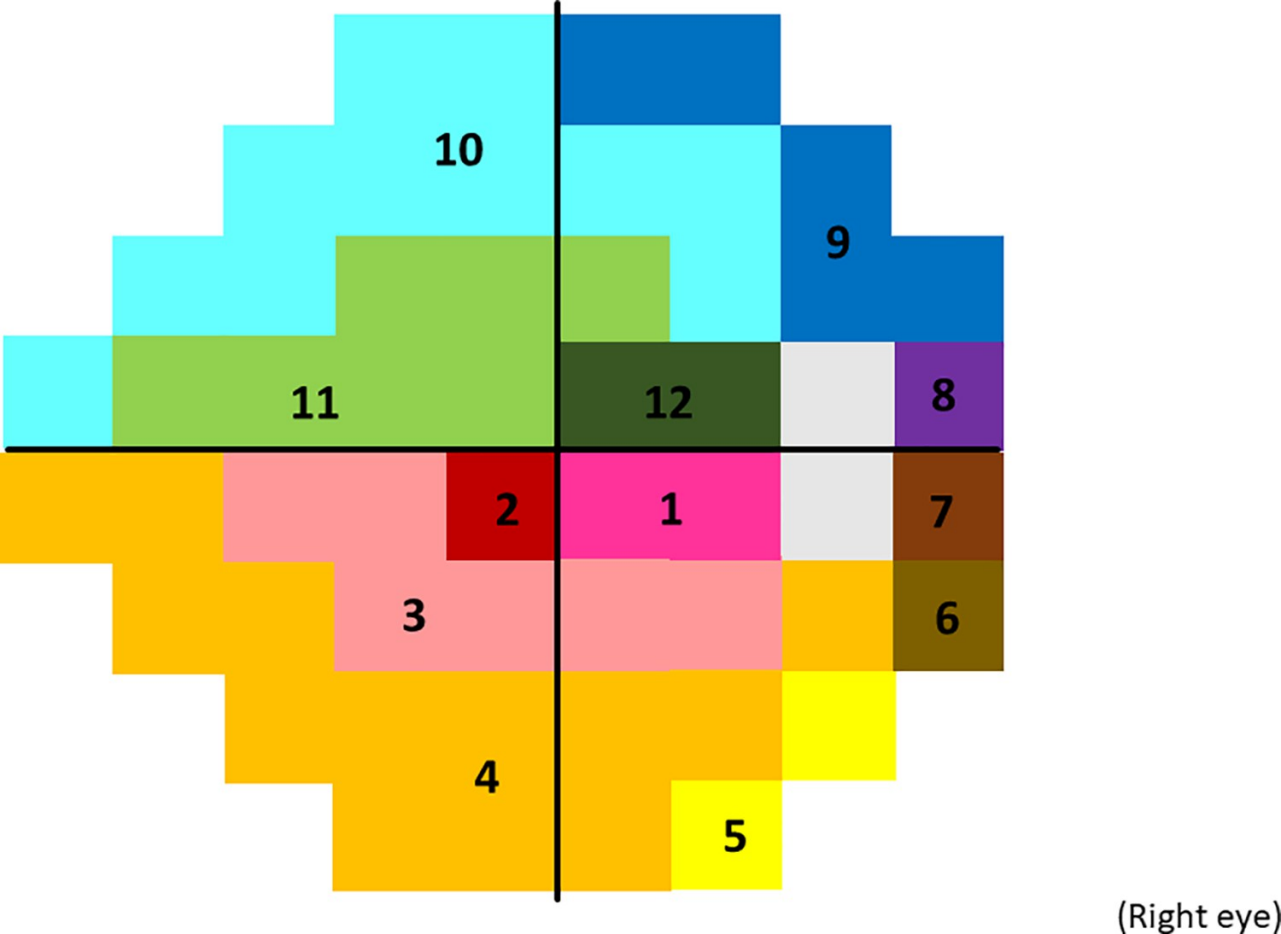
Twelve sectors correspond to 30 degrees optic disc angles. The visual field was divided into 12 sectors corresponding to 30 degrees of optic disc angles, based on the structure-function mapping by Garway-Heath et al. [18].

Finally, the VF was divided into 12 sectors corresponding to 12 cpRNFL optic disc sectors derived from the Garway-Heath structure-function map (**Fig 1**) [18]. Then the structure-function relationship was analyzed between pointwise visual sensitivity in a sector (fixed effect) and corresponding cpRNFL thickness measurements whereby the random effects were patients and test points (dependent variable was visual sensitivity), and compared between SITA standard and VBLR-VF.

All statistical analyses were carried out by using the statistical programming language R (version 3.1.3; The R Foundation for Statistical Computing, Vienna, Austria).

## Result

The background demographics of the studiesd 78 eyes of 56 patients are shown in **Table 1**. Of the 57 patients, 28 were men. Patients’ age was 61.0 ± 10.5 [40 to 84] (mean ± standard deviation, sd, [range]) y old. Out of the 78 eyes, 42 were right eyes. Out of 78 eyes, 70 were inherited from our previous study [7].

**Table 1:** Subjects’ demographics.

	mean	standard deviation
age, years	61.0	10.5
gender, male:female	28:28	
eye, right:left	42:32	
AL, μm	25.5	1.5
MD (SITA standard), dB	-7.4	6.2
PSD (SITA standard), dB	9.3	5
MD (VBLR VF), dB	-8.8	6.5
PSD (VBLR VF), dB	9.4	4.9
cpRNFL, μm	74.6	15.7

cpRNFL, circumpapillary retinal nerve fiber layer; MD, mean deviation; PSD, pattern standard deviation; SITA, Swedish interactive thresholding algorithm; VBLR, variational Bayes linear regression; VF, visual field.

The MD value with the SITA standard was −7.4 ± 6.2 (mean ± sd, [range: −19.5 to 3.4]) dB, whereas that with the VBLR-VF was −8.8 ± 6.5 [−23.1 to 1.4] dB. There was no significant difference between these MD values, although approached a significance (p = 0.066, linear mixed model). The PSD value with the SITA standard was 9.3 ± 5.0 (mean ± sd, [range: 1.1 to 17.2]) dB, whereas that with the VBLR-VF was 9.4 ± 4.9 [0.93 to 17.2] dB. There was no significant difference between these PSD values (p = 0.91). Test duration with SITA standard was 6 minutes and 4 seconds ± 1 minute and 7 seconds [3 minutes and 57 seconds to 8 minutes and 34 seconds], whereas that with VBLR-VF was 5 minutes and 30 seconds ± 1 minute and 30 seconds [2 minutes and 49 seconds to 8 minutes and 36 seconds]. There was a significant difference between these testing durations (*p* < 0.001).

**Fig 2:**
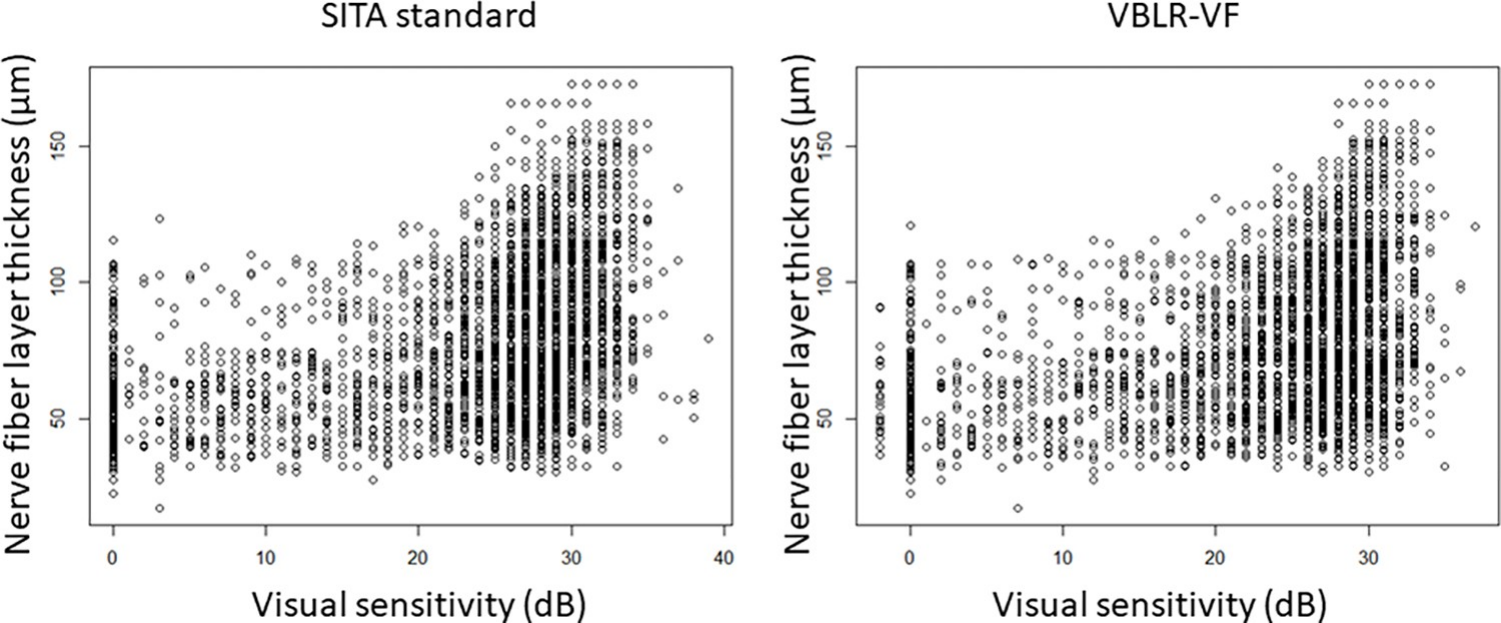
Relationship between visual sensitivity and cpRNFL (whole visual field). The relationship between visual sensitivity and cpRNFL in whole visual field is shown. cpRNFL, circumpapillary retinal nerve fiber layer, SITA, Swedish interactive thresholding algorithm; VBLR, variational Bayes linear regression; VF, visual field.

In the whole VF (**Fig 2**), the AICc values of SITA standard and VBLR were 601.6 and 597.3, respectively (linear mixed model, adjusted for age and axial length). The relative likelihood that VBLR had a better structure-function relationship than the SITA standard was 88.2%. Using all (52) test points, the AICc values between visual sensitivities and corresponding cpRNFL thicknesses were 36619.4 and 36608.5 with SITA standard and VBLR-VF (linear mixed model, adjusted for age and AL). The relative probability of the structure-function relation with VBLR-VF minimizes the information loss compared to the SITA standard was 99.9%.

**Fig 3:**
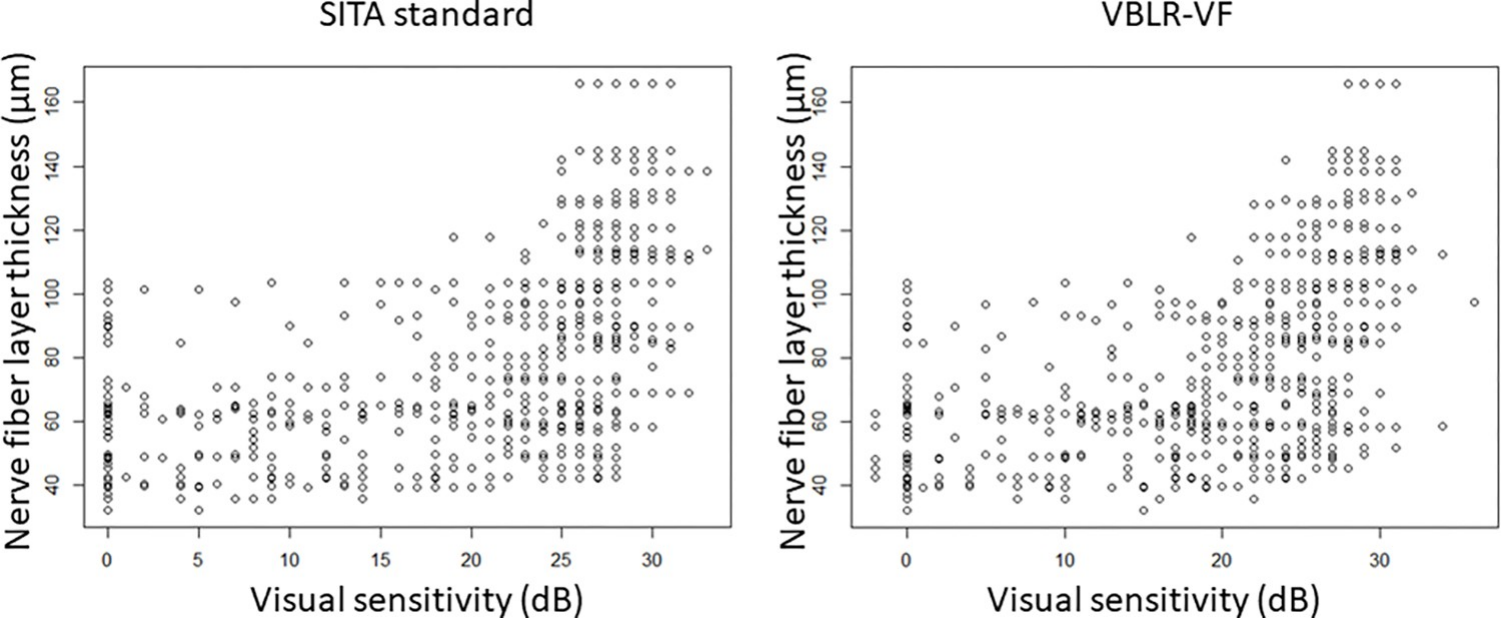
Relationship between visual sensitivity and cpRNFL (sector 10). The relationship between visual sensitivity and cpRNFL in sector 10 is shown. cpRNFL, circumpapillary retinal nerve fiber layer, SITA, Swedish interactive thresholding algorithm; VBLR, variational Bayes linear regression; VF, visual field.

**Table 2** shows the sectorial mean sensitivities with SITA standard and VBLR-VF. These values were lower with VBLR-VF compared to the SITA standard in 6 among 12 sectors (Sectors 3, 4, 7, 8, 11, and 12, p < 0.05, linear mixed model). **Table 3** shows the AICc values with SITA standard and VBLR-VF in each of the 12 sectors (**Fig 3** shows the scatter plot in sector 10). With the sector-wise analysis, SITA standard had a better structure-function relationship than VBLR in 1 sector (Superior sector in the retina) with >95% relative likelihood, whereas VBLR had a better structure-function relationship than SITA standard in 4 sectors (Supero-Nasal, Infero-Nasal, Inferior, and Infero-Temporal sectors) with >95% relative likelihood (linear mixed model, adjusted for age and AL).

**Table 2:** Average visual sensitivities and cpRNFL thickness in 12 sectors.

	SITA standard								VBLR-VF								p value	cpRNFL thickness		
sector1	29.4	±	5.9	[	0.0	to	37.5	]	29.2	±	4.8	[	0.0	to	34.0	]	0.62	65.1	±	16.6
sector2	27.7	±	9.4	[	0.0	to	36.0	]	28.0	±	8.9	[	0.0	to	35.0	]	0.29	75.4	±	26.3
sector3	24.3	±	9.8	[	0.0	to	33.8	]	23.5	±	9.8	[	0.0	to	32.5	]	**0.0053**	90.9	±	32.5
sector4	23.9	±	7.1	[	3.8	to	32.0	]	22.9	±	7.2	[	2.9	to	30.9	]	**<0.001**	86.5	±	26.8
sector5	27.5	±	3.8	[	13.5	to	35.0	]	26.9	±	3.5	[	13.0	to	31.0	]	0.10	91.4	±	23.1
sector6	27.4	±	3.3	[	18.0	to	33.0	]	26.8	±	3.1	[	9.0	to	31.0	]	0.12	72.9	±	17.3
sector7	27.4	±	4.4	[	3.0	to	38.0	]	26.2	±	5.5	[	0.0	to	33.0	]	**0.018**	59.0	±	13.6
sector8	27.2	±	4.1	[	0.0	to	36.0	]	25.8	±	6.6	[	0.0	to	31.0	]	**0.027**	61.9	±	14.6
sector9	20.9	±	7.7	[	0.0	to	30.8	]	20.5	±	8.3	[	0.0	to	31.0	]	0.23	84.3	±	21.0
sector10	17.1	±	9.9	[	0.0	to	30.5	]	16.6	±	9.9	[	0.0	to	31.1	]	0.14	75.8	±	31.4
sector11	15.6	±	11.8	[	0.0	to	32.6	]	14.8	±	###	[	0.0	to	32.0	]	**0.0017**	70.5	±	35.2
sector12	21.5	±	10.8	[	0.0	to	33.5	]	19.8	±	###	[	0.0	to	33.0	]	**0.0012**	61.2	±	20.8

P values in bold suggest <0.05.

cpRNFL, circumpapillary retinal nerve fiber layer; SITA, Swedish interactive thresholding algorithm; VBLR, variational Bayes linear regression; VF, visual field.

**Table 3:** Comparison of the values of AICc, mR^2^, and cR^2^, between SITA standard and VBLR-VF.

	SITA standard			VBLR VF			relative likelihood
	AICc	mR^2^	cR^2^	AICc	mR^2^	cR^2^	
sector1	1210.6	0.065	0.82	1213.2	0.59	0.81	0.73
sector2	709.1	0.26	0.55	711.4	0.91	0.93	0.69
sector3	3794	0.26	0.94	3791.2	0.84	0.87	0.76
sector4	7778	0.25	0.96	7789.9	0.73	0.79	**1**
sector5	1325.9	0.095	0.77	1317.6	0.15	0.72	**0.98**
sector6	665	0.095	0.77	661.5	0.16	0.72	0.82
sector7	636.2	0.022	0.17	637.6	0.32	0.55	0.51
sector8	646.7	0.026	0.27	648.1	0.25	0.26	0.52
sector9	2505.5	0.11	0.75	2485.9	0.75	0.81	**1**
sector10	7263.5	0.18	0.75	7207.9	0.77	0.81	**1**
sector11	5382.4	0.29	0.81	5358.7	0.85	0.87	**1**
sector12	1302.3	0.11	0.68	1307.3	0.79	0.83	0.92

The relative likelihood was calculated as exp((AIC*min* _ AIC*i*)/2).

AICc, second-order bias-corrected Akaike Information Criterion (AICc); SITA, Swedish interactive thresholding algorithm; VBLR, variational Bayes linear regression; VF, visual field, mR^2^: marginal R squared, cR^2^: conditional R squared.

## Discussion

In the current study, VF measurements were conducted using both SITA standard (HFA) and VBLR VF (KOA AP7700 perimetry) in 78 eyes of 56 patients with POAG, along with spectral-domain OCT measurement. Then structure-function relationship was investigated between visual sensitivity and cpRNFL in the whole VF. This analysis was iterated at each of 12 sectors (corresponds to 30 degrees sectors around the optic disc).

In this study, in the whole VF, the relative likelihood of VBLR had a better structure-function relationship than the SITA standard was 88.2% (when the entire field was averaged) or 99.9% (when all test points were analyzed in a pointwise manner). Sectorial analyses revealed that the SITA standard had a better structure-function relationship than VBLR in 1 in 12 sectors with >95% relative likelihood, whereas VBLR had a better structure-function relationship than the SITA standard in 4 sectors with >95% relative likelihood. These results suggest that VBLR-VF has no worse or better structure-function relationship than the SITA standard. The entire reason for this feasible result for VBLR-VF is not clear, however, it may be, due to the suppression of patient fatigue [19]. Even though these two algorithms have similar test-retest reproducibility, as suggested in our previous study [7], the clinical reliability of VBLR-VF is comparable to that of the SITA standard. Notably, our previous research found that the measurement duration with VBLR-VF was significantly shorter than the SITA standard [7]. The vast majority of the current patients were derived from a previous study (70 in 78 eyes), and as a result, a similar tendency was also observed in the current data. Both algorithms use the same 4–2 double cross staircase method, and hence this reduction of measurement duration is different from that between the SITA standard (4–2 double cross staircase) and SITA Fast 4 single cross staircase. In summary, these results suggested that VBLR-VF enabled a faster VF measurement without sacrificing the measurement reliability: test-retest reproducibility as shown in [7] and measurement accuracy as observed in the current study.

With sector-wise analysis, the structure-function relationship was similar and unlikely to make a clinically relevant difference between SITA standard and VBLR-VF, but the SITA standard had a better structure-function relationship than VBLR in 1 out of 12 sectors with >95% relative likelihood, whereas VBLR had a better structure-function relationship than the SITA standard in 4 sectors with >95% relative likelihood: see **Table 3**. More specifically, in sectors 10 and 11 (Inferior and Infero-Temporal sectors), the structure-function relationship was greater with VBLR-VF than with the SITA standard with >95% relative likelihood. These sectors may be the most appropriate regions to analyze the structure-function relationship in glaucoma because they are the most preferentially damaged in glaucomatous, and indeed, mean visual sensitivity was lowest in these sectors (see **Table 2**). In other sectors 1, 2, 3, 6, 7, 8, and 12 the difference in the structure-function relationship between the two algorithms did not have a relative likelihood >95%, which may be because of less obvious glaucomatous damage. A further investigation preparing more advanced cases in these regions would be needed to shed light on this issue.

The detection of progression relies on the frequency of VF measurement as well as the accuracy (variability) of VF, both of which are based on the results of a simulation experiment [20]. Based on the results of this experiment, it has been recommended sufficient number of VF measurements be performed to avoid delayed detection of progression, such as 6 tests in the first 2 years [21]. On the other hand, previous studies have revealed the difficulty of performing VF testing with sufficient frequency in busy clinics [22, 23]. The faster VF measurement with VBLR-VF than the SITA standard would be beneficial to approach the sufficient frequency, which would result in an improved detection ability of progression, albeit with the identical test-retest reproducibility [7] and no worse or better accuracy as shown in the current study, in the end.

This study has limitations. First, a similar comparison of structure-function should be conducted between VBLR-VF (4 dB single staircase) [8] and SITA Fast. Second, further validation would be needed for VBLR 10–2 VF test which is not available in AP-7700 perimetry. Third, a further investigation should be performed in terms of other aspects of clinical usefulness, such as the ability to detect progression in trends and also event analyses.

In conclusion, a comparison of the structure-function relationship in the current study revealed that, although it depends on locations, VBLR-VF had a better structure-function relationship than the SITA standard overall. Even though the two methods have similar test-retest reproducibility and a significantly shorter measurement duration [7], there is a clinical advantage to using VBLR-VF over the SITA standard.

## References

[1] Bengtsson B, Olsson J, Heijl A, Rootzen H. A new generation of algorithms for computerized threshold perimetry, SITA. Acta ophthalmologica Scandinavica. 1997;75(4):368–75. Epub 1997/08/01. doi: 10.1111/j.1600-0420.1997.tb00392.x . 9374242

[2] Bengtsson B, Heijl A. SITA Fast, a new rapid perimetric threshold test. Description of methods and evaluation in patients with manifest and suspect glaucoma. Acta ophthalmologica Scandinavica. 1998;76(4):431–7. Epub 1998/08/26. doi: 10.1034/j.1600-0420.1998.760408.x . 9716329

[3] Bengtsson B, Heijl A. Evaluation of a new perimetric threshold strategy, SITA, in patients with manifest and suspect glaucoma. Acta ophthalmologica Scandinavica. 1998;76(3):268–72. doi: 10.1034/j.1600-0420.1998.760303.x . 9686835

[4] Artes PH, Iwase A, Ohno Y, Kitazawa Y, Chauhan BC. Properties of perimetric threshold estimates from Full Threshold, SITA Standard, and SITA Fast strategies. Invest Ophthalmol Vis Sci. 2002;43(8):2654–9. Epub 2002/07/31. . 12147599

[5] Murata H, Zangwill LM, Fujino Y, Matsuura M, Miki A, Hirasawa K, et al. Validating Variational Bayes Linear Regression Method With Multi-Central Datasets. Invest Ophthalmol Vis Sci. 2018;59(5):1897–904. Epub 2018/04/21. doi: 10.1167/iovs.17-22907 ; PubMed Central PMCID: PMC5886131. 29677350 PMC5886131

[6] Murata H, Araie M, Asaoka R. A new approach to measure visual field progression in glaucoma patients using variational bayes linear regression. Invest Ophthalmol Vis Sci. 2014;55(12):8386–92. Epub 2014/11/22. doi: 10.1167/iovs.14-14625 . 25414192

[7] Murata H, Asaoka R, Fujino Y, Matsuura M, Hirasawa K, Shimada S, et al. Comparing the usefulness of a new algorithm to measure visual field using the variational Bayes linear regression in glaucoma patients, in comparison to the Swedish interactive thresholding algorithm. Br J Ophthalmol. 2022;106(5):660–6. Epub 20210113. doi: 10.1136/bjophthalmol-2020-318304 ; PubMed Central PMCID: PMC9046736. 33441321 PMC9046736

[8] Hirasawa K, Murata H, Shimada S, Matsuno M, Shoji N, Asaoka R. Faster algorithms to measure visual field using the variational Bayes linear regression model in glaucoma: comparison with SITA-Fast. Br J Ophthalmol. 2022. Epub 20220301. doi: 10.1136/bjophthalmol-2021-320523 . 35232725

[9] Murata H, Asaoka R, Fujino Y, Matsuura M, Hirasawa K, Shimada S, et al. Comparing the usefulness of a new algorithm to measure visual field using the variational Bayes linear regression in glaucoma patients, in comparison to the Swedish interactive thresholding algorithm. Br J Ophthalmol. 2021. Epub 2021/01/15. doi: 10.1136/bjophthalmol-2020-318304 . 33441321 PMC9046736

[10] Littman H. Zur Bestimmung der wahren Größe eines Objektes auf dem Hintergrund des lebenden Auges. Klin Monatsbl Augenheilkd. 1982;180:286–9. 7087358 10.1055/s-2008-1055068

[11] Littman H. Zur Bestimmung der wahren Größe eines Objektes auf dem Hintergrund eines lebenden Auges. Klin Monatsbl Augenheilkd 1988;192:66–7. 3352190 10.1055/s-2008-1050076

[12] Baayen RH, Davidson DJ, Bates DM. Mixed-effects modeling with crossed random effects for subjects and items. Journal of Memory and Language. 2008;59(4):390–412. doi: 10.1016/j.jml.2007.12.005

[13] Bates D, Mächler M, Bolker B, Walker S. Fitting Linear Mixed-Effects Models Usinglme4. Journal of Statistical Software. 2015;67(1). doi: 10.18637/jss.v067.i01

[14] Burnham KP AD. Multimodel inference: understanding: AIC and BIC in model selection. Sociological Methods & Research. 2004;33:261–304.

[15] Tibshirani RJ, Taylor J. Degrees of freedom in lasso problems. Annals of Statistics. 2012;40:1198–232.

[16] Mallows C. Some comments on Cp. Technometrics. 1973;15:661–75.

[17] Nakagawa S, Johnson PCD, Schielzeth H. The coefficient of determination R(2) and intra-class correlation coefficient from generalized linear mixed-effects models revisited and expanded. J R Soc Interface. 2017;14(134). Epub 2017/09/15. doi: 10.1098/rsif.2017.0213 ; PubMed Central PMCID: PMC5636267. 28904005 PMC5636267

[18] Garway-Heath DF, Poinoosawmy D, Fitzke FW, Hitchings RA. Mapping the visual field to the optic disc in normal tension glaucoma eyes. Ophthalmology. 2000;107(10):1809–15. doi: 10.1016/s0161-6420(00)00284-0 . 11013178

[19] Johnson CA, Adams CW, Lewis RA. Fatigue effects in automated perimetry. Appl Opt. 1988;27(6):1030–7. Epub 1988/03/15. doi: 10.1364/AO.27.001030 . 20531515

[20] Jansonius NM. On the accuracy of measuring rates of visual field change in glaucoma. Br J Ophthalmol. 2010;94(10):1404–5. Epub 20100615. doi: 10.1136/bjo.2009.164897 . 20554508

[21] Chauhan BC, Garway-Heath DF, Goni FJ, Rossetti L, Bengtsson B, Viswanathan AC, et al. Practical recommendations for measuring rates of visual field change in glaucoma. Br J Ophthalmol. 2008;92(4):569–73. Epub 20080122. doi: 10.1136/bjo.2007.135012 ; PubMed Central PMCID: PMC2564806. 18211935 PMC2564806

[22] Malik R, Baker H, Russell RA, Crabb DP. A survey of attitudes of glaucoma subspecialists in England and Wales to visual field test intervals in relation to NICE guidelines. BMJ Open. 2013;3(5). Epub 20130503. doi: 10.1136/bmjopen-2012-002067 ; PubMed Central PMCID: PMC3646174. 23645919 PMC3646174

[23] Crabb DP, Russell RA, Malik R, Anand N, Baker H, Boodhna T, et al. Frequency of visual field testing when monitoring patients newly diagnosed with glaucoma: mixed methods and modelling. Health Services and Delivery Research. Southampton (UK)2014. 25642569

